# Identifying discriminant factors between phantom limb pain, residual limb pain, and both in people with lower limb amputations: a cross-sectional study

**DOI:** 10.1097/MRR.0000000000000634

**Published:** 2024-07-15

**Authors:** Sanaz Pournajaf, Carlo Damiani, Francesco Agostini, Giovanni Morone, Stefania Proietti, Roberto Casale, Marco Franceschini, Michela Goffredo

**Affiliations:** a Research Area in Neuromotor Rehabilitation and Rehabilitation Robotics, Department of Neurosciences, IRCCS San Raffaele; b Department of Anatomical and Histological Sciences, Legal Medicine and Orthopedics, Sapienza University of Rome, Rome; c Department of Life, Health and Environmental Sciences, University of L’Aquila, Aquila; d San Raffaele Institute of Sulmona, Sulmona; e Opusmedica, NOP Persons Care & Research, Piacenza; f Department of Human Sciences and Promotion of the Quality of Life, San Raffaele Open University, Rome, Italy

**Keywords:** lower limb amputation, pain, phantom limb pain, rehabilitation, residual limb pain, stump pain

## Abstract

Postamputation pain is a common condition in patients with lower limb amputation (LLA), which compromises amputees’ rehabilitation, use of the prosthesis, and quality of life. The aim of our study was to investigate the prevalence of phantom limb pain (PLP), residual limb pain (RLP), or both types of pain among individuals with LLA, and to identify the factors associated with the presence of one type of pain versus the other. Patients who underwent amputation for traumatic or vascular reasons and who reported on RLP or PLP were analyzed and divided into three groups: PLP, RLP, or a group of subjects that presented both pains. We searched for factors that affect the occurrence of limb pain using univariate analyses, followed by multinomial logistic regression. Among the 282 participants with transtibial and transfemoral amputations, 192 participants (150 male and 42 female) presented PLP, RLP, or both types of pain, while 90 participants declared to perceive no pain. The estimated prevalence of any type of pain after transfemoral and transtibial amputation was therefore 68% (27% PLP, 10% RLP, and 31% both). Among the studied characteristics, only amputation level was associated with the type of pain (*P* = 0.001). Multinomial logistic regression identified transfemoral amputation as the only statistically significant predictor for PLP (odds ratio = 2.8; *P* = 0.002). Hence, it was estimated that individuals with transfemoral amputation have nearly three times higher odds of experiencing PLP compared with those with transtibial amputation.

## Introduction

Beyond the challenges related to functional impairments, individuals undergoing life-altering lower limb amputation (LLA) surgery may experience different types of pain, which they may localize to the residual leg segment or perceive as originating from the amputated leg segment [1]. Residual limb pain (RLP), also known as stump pain, manifests at the surgical site or proximal remaining extremity portion of the limb postamputation and can stem from various physical impairments such as skin conditions, vascular abnormalities, the impaired healing process, neuromas, soft tissue, and bone disorders, or complications related to prosthesis adaptation [2]. Conversely, phantom limb pain (PLP) presents as a chronic neuropathic pain sensation originating from the missing limb, driven by neuroplastic changes within the peripheral and central nervous systems [3,4]. Described as any unpleasant sensation, movement, or posture perceived in the absent body part, PLP prevalence ranges between 50 and 85.6% in existing literature [5]. While it is acknowledged that PLP may decrease over time, its persistence beyond 6 months is frequently associated with a less favorable prognosis for subsequent reduction [6–8]. It is worth noting that while both types of pain may occur simultaneously, complicating the condition’s management, RLP is typically more prevalent in the immediate postoperative phase, whereas PLP tends to manifest later and persist for longer periods. Additionally, RLP typically exhibits a more acute onset following surgery and demonstrates greater improvement over time compared with PLP [2].

Presently, the precise pathophysiology remains poorly understood, presumed to be multifactorial. Although drug therapy remains the predominant treatment option despite the condition’s inadequate management, the recognition of the necessity for nondrug interventions has grown [9,10]. Nonpharmacological treatments such as Transcutaneous Electrical Nerve Stimulation, mirror therapy, deep brain stimulation, acupuncture, relaxation, and biofeedback have been acknowledged, with some demonstrating more specific and effective management for PLP, while others for RLP [9–11].

In the context of rehabilitation after LLA, the presence of pain and its main types hold significant importance due to their implications for functioning, quality of life postsurgery, and patient compliance with prosthetic use, but also to address a targeted treatment approach [12–14]. Recent rehabilitative approaches for people with amputation tend to offer targeted rehabilitation, including specific pain management. Recent literature highlights the positive effect of rehabilitation methods to reduce pain management, particularly PLP in persons with LLA, such as mirror therapy, phantom motor execution, immersive and nonimmersive therapy, or a combination of these approaches [15–17]. Additionally, RLP management can benefit from nonsurgical approaches such as desensitization procedures including gentle massage, light tapping, vibration, constant pressure, and the application of various fabrics to the sensitive area, as well as Transcutaneous Electrical Nerve Stimulation, stretching exercises, and wound treatments [18].

Existing research predominantly focuses on prognostic factors associated with PLP, often overlooking the distinctions between RLP and PLP, as well as neglecting to explore the combined impact of experiencing both types of pain simultaneously on functional status and disability levels within this population [19]. On the other hand, the projected doubling of the population living with limb loss in the USA by 2050 underscores the importance of obtaining comprehensive insights into the prevalence of postamputation pain and its subtypes [20]. Such understanding empowers clinicians, researchers, and policymakers to effectively allocate healthcare resources in anticipation of future demands [21]. With these considerations in mind, our study was designed to provide insights into the prevalence of specific types of pain in a relatively large sample of individuals with LLA. Our hypothesis was that demographic and clinical characteristics may serve as indicators of the likelihood of experiencing a specific type of pain, thereby suggesting targeted decision-making in postamputation pain management. Thus, the aim of our study was to investigate the prevalence of RLP, PLP, or both types of pain among individuals with LLA, and to identify the factors associated with the presence of one type of pain versus the other.

## Materials and methods

This secondary analysis, cross-sectional study was conducted on a database of 687 individuals with LLA who received a prosthesis with the same fabrication and underwent homogeneous clinical optimization of the prosthesis itself in addition to a rehabilitation treatment using a standard rehabilitation protocol [22].

The database was applied in this study compatibly with the privacy guarantee regulations (Official Gazette No. 190 of 08/14/2008).

The telephone survey was based on a questionnaire that explored the socio-demographic and general clinical status before and after the amputation. The following factors were identified as independent variables, therefore as prognostic indicators: age, gender, level of amputation, side of amputation, cause of amputation, time since last LLA, pre- and post-LLA working status (yes/no), current operating status (yes/no), and use of the prosthesis (yes/no). Also, clinical information regarding the presence or absence of RLP, PLP, and both types of pain and their intensity assessed by the Numeric Rating Scale (NRS) [23], the level of functional independence assessed by the Modified Rankin Score (MRS) [24], and autonomy and participation according to the Walking Handicap Scale (WHS) [25]. The MRS is a widely used single-item, global outcomes rating scale employed in assessing activity limitations in poststroke individuals. It serves as a straightforward tool for categorizing the level of functional independence based on precondition activities rather than specific task performance [24]. Typically, the MRS is administered through a guided interview with the patient, during which they are queried about their activities of daily living, encompassing both indoor and outdoor tasks [26]. While the applicability and validity of the MRS in the context of individuals with LLA may not be as firmly established as in stroke patients, there is potential for its adaptation in the amputee population. The WHS is a validated assessment tool designed to evaluate mobility and participation utilized across various conditions, including individuals with LLA [22]. It typically assesses various aspects related to walking ability, including the use of assistive devices, balance, endurance, and the individual’s ability to perform mobility-related activities in different environments (e.g. indoors, outdoors, on different surfaces) [25].

We included persons with unilateral transtibial or transfemoral LLA from traumatic or vascular origin, aged between 18 and 80 years, referred to the same Orthopedic Prosthesis Center for LLA prostheses and underwent clinical optimization of the prosthesis in the last decades, from 2017 to today. Informed consent to participate in the project and to answer a telephone survey was obtained in writing. Subjects with neoplastic origin of LLA, upper limb or lower bilaterally limb amputation, different types of amputation from transtibial and transfemoral, incomplete data available in the database, clinical implications in the first year after the last wound-related surgery postoperative, and lack of informed consent were excluded [22].

**Fig. 1:**
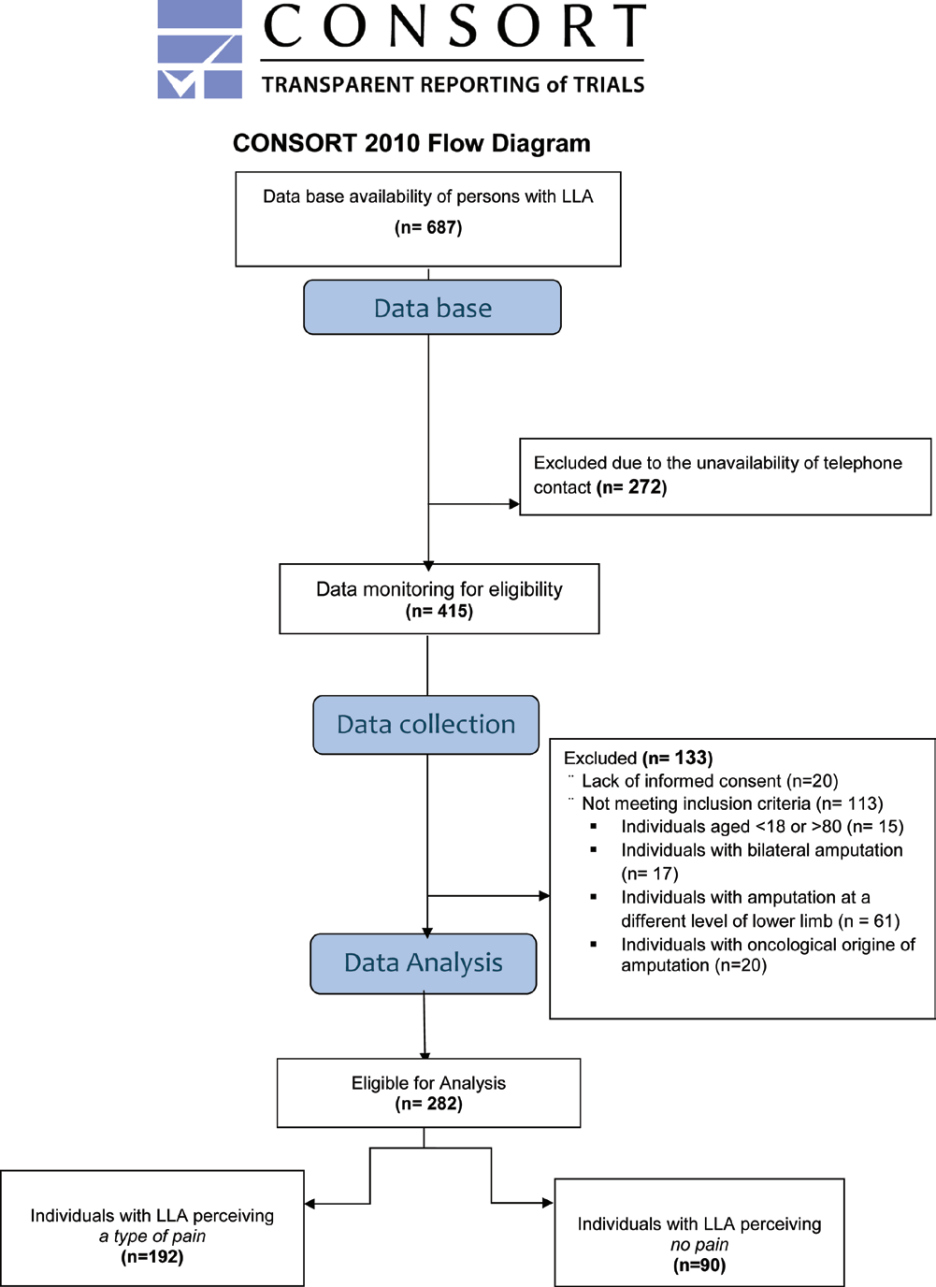
Study flow chart.

In total, 282 subjects satisfied the inclusion/exclusion criteria (Fig. 1 flow chart). The individuals included were surveyed via telephone by a trained psychologist, who was unaware of the purpose of the study and was not involved in the data processing, through a structured questionnaire decided with the medical team (please see questionnaire in Supplementary Material, Supplemental digital content 1, http://links.lww.com/IJRR/A51).

The study was approved by the local ethics committee on 08 July 2018, with number 07/2018.

### Statistical analysis

Statistical analyses were performed using IBM SPSS Statistics, version 28.0 (SPS s.r.l.; Bologna, Italy). Descriptive statistics were used to summarize and describe the continuous variables: mean and SD, if normally distributed, otherwise expressed as median and interquartile range; for categorical variables, frequency (count) and percentage were used. In bivariate analysis, the chi-square test was applied if the comparison involved categorical data, while the analysis of variance was applied to compare quantitative data against a factor, testing for the existence of significant differences in means among groups. In the univariate analysis, Bonferroni’s correction for multiple comparisons was applied to the data from the rating scales. The multinomial logistic regression was employed as the multivariate analysis technique to model nominal outcome variables because the type of pain was a nominal variable with three categories. The variables that were significant in the univariate analysis were included in the multivariate analysis. The group with both types of pain was considered as the reference category. The coefficients obtained from the regression analysis represented the effect of the independent variables on the log odds of belonging to each category with respect to the reference category. All variables were considered statistically significant at *P*-value less than 0.05.

## Results

From a sample of 687 records on persons with amputation, 415 individuals were considered potentially eligible for the study; 133 persons were excluded because they did not meet the eligibility criteria due to different reasons, such as lack of consent to participate in the study, age range, level, and cause of the amputation. A total of 282 individuals with LLA were thus considered for the descriptive analysis. The second phase of the analysis was carried out on a total of 192 patients (150 male and 42 female), excluding 90 participants who did not declare to perceive any type of pain (neither RLP nor PLP; Fig. 1). The characteristics of the included sample are shown in Table 1. The prevalence rate of any type of pain (PLP, RLP, both) in our selected sample having a transfemoral and transtibial amputation (*N* = 282) was 68% (27%, 10%, and 31%, respectively).

**Table 1:** **Characteristics of the sample included (*N* = 282**)

Gender, *n* (%) Male Female	226 (80) 56 (20)
Type of pain, *n* (%) RLP PLP Both No pain	29 (10) 77 (27) 86 (31) 90 (32)
Amputation level, *n* (%) TT TF	140 (50) 142 (50)
Cause, *n* (%) Traumatic Vascular	143 (51) 139 (49)
Side, *n* (%) Right Left	126 (45) 156 (55)
Age (y.o.), mean± SD; *n* (%) 18–50 51–70 >71	60.8 ± 14.1 65 (23) 126 (45) 91 (32)
Time since last LLA (years), *n* (%) 1–2 3–5 6–8 +9	172 (61) 39 (14) 20 (7) 50 (18)
NRS, *n* (%) 0 1–3 4–7 8–10	90 (31) 13 (5) 100 (36) 76 (28)
WHS, *n* (%) 1–3 4–6	76 (27) 206 (73)
MRS, *n* (%) 1–3 4–5	129 (46) 153 (54)
Use of prothesis, *n* (%) Yes No	225 (80) 57 (20)

LLA, lower limb amputation; MRS, Modified Rankin Score; NRS, Numeric Rating Scale; PLP, phantom limb pain; RLP, residual limb pain; TF, transfemoral; TT, transtibial; y.o., years old; WHS, Walking Handicap Scale.

The model showed that only amputation level as the independent variable was statistically significant in predicting the probability of the type of pain; sex, age, time since last LLA, cause of amputation, and use of the prosthesis were not statistically significantly associated with the pain type.

This indicates that these variables do not significantly affect the pain response. Furthermore, there was no statistically significant difference between the three groups in pain rating scales (NRS, MRS, and WHS) (Table 2). Regarding the clinical evaluation scales (NRS, MRS, and WHS), it was observed that pain intensity, functional status, and disability were not statistically significantly associated with the perception of pain type (Table 2); while the comparison of pain types (PLP, RLP, or both) in relation to the level of amputation (transtibial and transfemoral) yielded significant findings (*P* = 0.001) (Table 3).

**Table 2:** **Univariate analysis (*N* = 192**)

Independent variables	Dependent variables Types of pain			
	PLP	RLP	Both	*P* value
Gender, n (%) Female Male	17 (22) 60 (78)	3 (10) 26 (90)	22 (26) 64 (74)	0.189
Age (y.o.), mean (SD)	62.4 (12.5)	59.6 (16.1)	61.2 (13.6)	0.633
Time since last LLA (years), mean (SD)	1.6 (1.0)	2.3 (1.9)	2.0 (1.7)	0.045
Side, *n* (%) Right Left	39 (51) 38 (49)	11 (38) 18 (62)	40 (46) 46 (54)	0.502
Cause, *n* (%) Traumatic Vascular	28 (36) 49 (64)	16 (55) 13 (45)	41 (48) 45 (52)	0.153
Use of prothesis (yes), *n* (%)	62 (80)	24 (83)	59 (69)	0.130
NRS, mean (SD)	6.7 (2.3)	6.4 (2.0)	6.9 (2.0)	0.567
MRS, mean (SD)	3.2 (1.3)	2.7 (1.6)	3.1 (1.5)	0.303
WHS, mean (SD)	4.0 (1.9)	4.1 (2.1)	3.7 (2.1)	0.619

LLA, lower limb amputation; MRS, Modified Rankin Score; NRS, Numeric Rating Scale; PLP, phantom limb pain; RLP, residual limb pain; y.o., years old; WHS, Walking Handicap Scale.

**Table 3:** **Univariate analysis (*N* = 192**)

Independent variables	Dependent variables Types of pain		
	PLP	RLP	Both
	*χ*^2^, *P* value		
Gender	2.949, 0.229		
Age	2.931, 0.569		
Cause	3752, 0.153		
Side	1.377, 0.502		
Amputation level	13.016, 0.001		
Use of prothesis	4.088, 0.130		
NRS, *n* (%)	1.614, 0.806		
WHS, *n* (%)	1.505, 0.471		
MRS, *n* (%)	1.287, 0.525		

MRS, Modified Rankin Score; NRS, Numeric Rating Scale; PLP, phantom limb pain; RLP, residual limb pain; y.o., years old; WHS, Walking Handicap Scale.

Multinomial logistic regression was utilized to model the nominal outcome variable (having three possible values: PLP, RLP, or both), where the logarithmic odds of the outcomes are represented as a combination of predictive variables. In this study, the model included the level of amputation (as it was the only significant variable identified in the univariate analysis) and age category. The results of the multinomial logistic regression indicate that the overall model is significant, with *χ*^2^ = 15.535; *P* = 0.016.

However, transfemoral (as compared to transtibial) amputation was only a statistically significant predictor of experiencing PLP (as opposed to experiencing both types of pain). It was estimated that individuals after transfemoral amputation have 2.7 times higher odds of experiencing PLP rather than both types of pain compared to those after transtibial amputation (Table 4).

**Table 4:** **Summary of multinomial logistic regression (*n* = 192**)

Type of pain		Beta	Standard error	Wald’s test	Degrees of freedom	*P* value	OR	95% CI	
								Lower	Upper
PLP vs. both	Amp. level TF vs. TT	1.020	0.336	9.202	1	**0.002**	2.772	1.435	5.357
	18–50 vs. >71 years	0.108	0.475	0.052	1	0.820	1.114	0.439	2.827
	51–70 vs. >71 years	0.347	0.368	0.887	1	0.346	1.414	0.687	2.910
RLP vs. both	Amp. level TF vs. TT	−0.284	0.448	0.402	1	0.526	0.752	0.312	1.812
	18–50 vs. >71 years	0.525	0.582	0.816	1	0.366	1.691	0.541	5.287
	51–70 vs. >71 years	0.069	0.522	0.017	1	0.895	1.071	0.385	2.983

Significant *P* values are reported in bold.

PLP, phantom limb pain; RLP, residual limb pain; TF, transfemoral; TT, transtibial.

## Discussion

This cross-sectional study, involving 282 participants, aimed to investigate the prevalence of RLP, PLP, or both types of pain among individuals with LLA, as well as to identify the factors associated with the presence of one type of pain versus the other, considering only the sample of persons experiencing a type of pain (*N* = 192). Understanding the implications of pain types for functioning, quality of life postsurgery, and patient compliance with prosthetic use is crucial, emphasizing the need for targeted treatment approaches [12–14]. Our local telephone survey sought to address this gap in research by examining the prevalence and factors associated with different types of pain in a relatively large sample of individuals with LLA. Predicting pain type is essential for anticipating tailored treatment approaches, ensuring earlier and more effective pain management.

The prevalence of the PLP observed in our cohort of individuals with LLA was 55.0% (both alone and in combination with RLP). This result is slightly lower if compared with the results obtained in a recent review that included more than 12 000 individuals with amputation. However, the 64% of PLP prevalence estimation in this study took into consideration all kinds of amputation (upper limb and lower limb) [5]. Conversely, the prevalence of the RLP alone was the lowest one in our selected sample (10%) in line with the literature, in relation to its time since the last LLA (2.3 ± 1.9), which reports a prevalence varying from 10 to 13% at 2 years postamputation to 55–76% in longstanding amputees [27]. On the other hand, among the 32% of the sample not experiencing any type of pain (*N* = 90), 72% were more than 3 years removed from their last LLA, thus confirming in part the literature suggesting that pain relief can occur progressively, particularly in case of individuals suffering PLP [28]. Nevertheless, it is essential to acknowledge a limitation of our survey: we did not inquire about the prior pain experiences of these individuals, which could have provided valuable insights. Our results demonstrated that subjects with transfemoral amputation have a higher probability (almost three times) to experience PLP than those with transtibial amputation. This result is in agreement with the literature that identified the proximal site of amputation as a risk factor for PLP [5]. In line with this result, there were also other studies that found a higher prevalence of the PLP in subjects with shorter residual limbs [29] and in above knee amputations [30].

We have observed a high incidence (31%) of persons with LLA not using prosthesis in the group with both types of pain perceiving a mild to severe pain intensity (NRS = 5.0 ± 3.6) with respect to the other two groups with PLP or RLP alone (20 and 17%, respectively). However, it seems that the lack of prosthesis use and high pain intensity in this group did not affect their level of functional independence, mobility, and participation (MRS and WHS, respectively) compared with the other two groups experiencing only one pain type alone. This is in line with literature that shows no correlation between pain intensity and community amputation regardless of its subtype [22]. Additionally, the time elapsed since the last LLA was not a significant indicator for the type of postamputation pain. While there was a trend toward significance, possibly due to the relatively small sample size, it is worth noting. If this trend is confirmed in future studies, it may indicate that there is a higher prevalence of PLP in the first year postamputation, which tends to decrease over time, while residual limb pain RLP proportionally increases. This initial observation is supported by existing literature, which shows that PLP gradually reduces over time, even beyond the first year [28]. Contrarily, an American national survey conducted from 1998 to 2000 by Ephraim *et al*., [31] has also found a high prevalence of all types of chronic amputation-related pain regardless of time since amputation in people with limb loss, considering both upper and lower limb amputees. The authors have also found the presence of depressive symptoms as a predictor of an increased level of pain intensity and bothersomeness suggesting the need to elucidate the relationship between pain and depressive symptoms among this population [31]. This remains a limit of our study that did not include details on the psychological state of the participants.

Yerli *et al*. [32] found that phantom pain and stump pain in below-knee amputees limited activity and participation, with phantom pain having a more negative effect on activity levels than stump pain [32]. In contrast, our study did not reveal a significant correlation between activity and participation limitations assessed, respectively, by the MRS and WHS, indicating a lack of predictive power for perceiving one type of pain versus another in individuals with LLA. It is important to note that while the MRS may offer insights into the functional independence of individuals with LLA during daily activities, its applicability in this population may require careful validation to ensure relevance and accuracy. The specific domains assessed by the MRS may not fully capture the unique functional status and disability profile of individuals with lower limb amputations, which may have influenced our results and should be considered a limitation of our study. Therefore, further research and validation studies are warranted to assess the suitability of the MRS for evaluating the functional status and disability in individuals with lower limb amputations.

An important aspect of our current study involves the investigation and differentiation of RLP, which was observed in 41% of the participants, either independently or in conjunction with PLP. RLP and PLP represent distinct clinical entities; however, RLP may serve as a trigger for PLP. Hence, an accurate and timely diagnosis is crucial. Moreover, timely diagnosis is a prerequisite for personalized management, enabling early intervention to mitigate the impact on the patient’s functionality and quality of life.

### Conclusion

Clinicians should be cognizant of the prevalence and impact of both PLP and RLP or both conditions coexisting simultaneously in lower limb amputee patients. Implementing appropriate strategies to mitigate these pains, including addressing known risk factors, is paramount. Furthermore, recognizing and distinguishing between these distinct clinical pain entities is essential for tailoring pharmacological and rehabilitation treatments effectively. It is crucial to note that individuals affected by transfemoral amputation have a 2.7 times higher probability of experiencing PLP.

## Acknowledgements

The research activity of S.P., C.D., F.A., S.P., M.F., and M.G. has been funded by the Italian Ministry of Health [Ricerca Corrente].

The study was approved by the local Ethics Committee on 08 July 2018 with the number 07/2018.

Written informed consent was obtained from all subjects before the study.

### Conflicts of interest

There are no conflicts of interest.

## Supplementary Material


