# Supplementary material for: Identifying discriminant factors between phantom limb pain, residual limb pain, and both in people with lower limb amputations: a cross-sectional study
Source: Int J Rehabil Res. 2024 Jul 15;47(3):214–20. doi: 10.1097/MRR.0000000000000634 (PMC11424063; doi:10.1097/MRR.0000000000000634)
Supplement: Supplementary file 1 [file ijrr-47-214-s001.pdf]

Supplemental Digital Content (Appendix 1)

Participation/Activity Questionnaire for people with Lower Limb Amputation

GENERAL DATA

Date of survey :

Patient's Identification Number (ID) :

Last name : First name :

Address :

Contact information (Tel/Cell. Number) :

DEMOGRAPHIC DATA

Date of birth/age :

Gender: Female ☐ Male ☐

Dominant hand: Right Left ☐

ANAMNESIS

Date of last amputation (or period):

Etiology (cause of amputation):

Bilateral:	Yes ☐	No ☐	
Amputation Side:	Right ☐	Left ☐	
Amputation Level:	Trans-Femoral ☐	Trans-Tibial ☐	Other ☐
Single Surgery:	Yes ☐	No ☐	

Other associated pathologies:

SOCIAL DATA

Work activity before last amputation: Yes ☐ No ☐

Current work activity: Yes ☐ No ☐

Do you need help to carry out your daily activities (personal hygiene, dressing, etc)?

Yes NO

How much assistance do you need?

- Maximum ☐
- Moderate ☐
- Consistent ☐

Do you also need assistance out of home? Yes ☐ No ☐

If yes, please choose one of the items. Only supervision ☐ Constant assistance ☐

Do you walk? Yes ☐ No☐

Are you able to walk indoor? ☐

Are you able to walk outdoor? ☐

With assistance? ☐

Without assistance? ☐

Do you feel pain at the amp. limb? Yes ☐ No ☐

Which kind of pain?

Phantom Limb Pain ☐

Stump Pain ☐
